# “Until the trial is complete you can’t really say whether it helped you or not, can you?”: exploring cancer patients’ perceptions of taking part in a trial of acupressure wristbands

**DOI:** 10.1186/1472-6882-13-260

**Published:** 2013-10-08

**Authors:** John Gareth Hughes, Wanda Russell, Matthew Breckons, Janet Richardson, Mari Lloyd-Williams, Alex Molassiotis

**Affiliations:** 1 Royal London Hospital for Integrated Medicine, UCLH NHS Trust, 60 Great Ormond Street, WC1N 3HR London, UK; 2 School of Nursing, Midwifery & Social Work, University of Manchester, Manchester, UK; 3 Institute of Health & Society, Newcastle University, Newcastle, UK; 4 Faculty of Health, Education and Society, University of Plymouth, Plymouth, UK; 5 Academic Palliative and Supportive Care Studies Group, Division of Primary Care, University of Liverpool, Liverpool, UK

**Keywords:** Cancer, Chemotherapy, Nausea, Vomiting, Acupressure, Acupressure Wristbands, Qualitative research

## Abstract

**Background:**

Nested qualitative studies within clinical trials provide data on patients’ experiences of receiving trial interventions and can inform and improve trial designs. The present study explored patients’ experiences of participating in a randomised controlled trial of acupressure wristbands for chemotherapy related nausea.

**Methods:**

A randomised three-group sham-controlled trial was carried out to evaluate the effectiveness of acupressure wristbands in the management of chemotherapy-related nausea. A convenience sample of 26 patients volunteered to participate in a qualitative study to explore their experiences of using acupressure wristbands, and taking part in the clinical trial. Participants were recruited from each of the three UK geographical sites from which the trial was conducted: Manchester, Liverpool and Plymouth. In-depth semi-structured interviews were conducted with the participants in their own homes or other location convenient for participating patients. Interviews were audio-taped, transcribed verbatim and analysed using Framework methodology.

**Results:**

The main motivational factors influencing participants to take part in the trial were a desire to 'give something back’ and limit their own experience of nausea. Participants were largely satisfied with the organisation and running of the acupressure wristband trial. Many participants experienced positive outcomes as a result of taking part in the trial. Lapses in memory, or poor health as a result of their chemotherapy treatment, led to some participants failing to complete trial paperwork on designated days. Two sham wristband participants reported wearing the bands inappropriately resulting in pressure being applied to the acupoint. Almost all of the participants interviewed had only experienced mild nausea or vomiting during the trial. Participants were pragmatic on the extent to which the wristbands were responsible for this lack of nausea and vomiting during the trial. However, many participants, including some patients receiving sham acupressure, believed the wristbands to have had a positive impact on their nausea and vomiting; there was a perception that the wristbands were, at least in part, responsible for the lack of nausea and vomiting they had experienced.

**Conclusions:**

Participants perceive acupressure wristbands as reducing the level of nausea and vomiting experienced during chemotherapy treatment. Reports that some participants wore wristbands inappropriately, and/or delayed completion of trial paperwork could represent confounding variables and have implications for the trial results, and the design of clinical trials within the field of cancer.

## Background

Qualitative research has been used extensively to explore patients’ experiences of complementary and alternative medicine (CAM) including acupuncture [1-6]. Findings suggest that acupuncture treatment frequently produces benefits beyond the alleviation of the patients’ presenting condition. These co-benefits include improvements in physical/mental health, emotional well-being and changes in personal identity and lifestyle, and can result in patients 'feeling normal again’ and 'regaining their lives.’ There is however a lack of research which has explored the experiences of users of acupressure or acupressure wristbands. Furthermore, although the concept of 'nested qualitative studies’ in the context of randomised clinical trials (RCTs) is now commonplace in studies evaluating conventional treatments, this is notably absent from CAM studies.

Qualitative studies nested within an RCT can inform and improve trial designs. Published research has explored aspects of trial participation, including recruitment, lay understandings of randomisation, informed consent, treatment compliance and retention to the trial [7-11]. Additionally, they can provide valuable data on patients’ experiences of receiving trial interventions and their perceptions of treatment effects (which may differ from those of patients receiving the same treatment within clinical practice). Trial context will have an impact on how treatment is perceived; participants in a clinical trial will have to read patient information sheets, be randomised to receive an active or placebo intervention, and complete trial paperwork throughout the length of their participation.

A search of the literature reveals a limited number of nested qualitative studies exploring patients’ experiences of participating in trials of CAM interventions. These studies have been nested within clinical trials of acupuncture for medically unexplained symptoms, osteoarthritis, migraine, irritable bowel syndrome and stress [12-16]. All explored patients’ experiences of receiving treatment within the context of a clinical trial, however two additionally explored patients’ experiences of taking part in the trial itself [14,16]. Paterson et al. conducted a study within a randomised sham controlled trial of traditional Chinese acupuncture for patients with migraine [16]. Patients in the Paterson et al. study were found to have actively 'played their part’ in the trial, taking on research roles that differed from their usual role as patients. The authors suggested that the participation in the trial resulted in changes to the normal expectations and behaviour and this changed how the intervention was experienced.

This paper reports on a qualitative study that was nested within a randomised, single-blind, sham-controlled trial of acupressure wristbands for chemotherapy related nausea. The research aimed to explore their experiences of using acupressure wristbands and taking part in a randomised controlled trial.

### Methodology

This qualitative study was nested within a RCT of acupressure wristbands for chemotherapy related nausea [17]. The study was carried out at three geographical sites within the UK: Manchester, Liverpool, Plymouth and the surrounding regions. Interviews took place between September 2010 and March 2011. Ethical approval for both the acupressure wristband trial and the nested qualitative study were obtained from Central Manchester Research Ethics Committee [REC reference number: 08/H1008/2]. The acupressure wristband trial is registered with the ISRCTN register [number ISRCTN87604299].

### The trial

A randomised, single-blind, sham-controlled, three armed trial was carried out to evaluate the effectiveness of acupressure wristbands for chemotherapy-related nausea [17]. 500 patients with heterogeneous cancer diagnoses receiving low, moderate and highly emetogenic chemotherapy were included in the trial. Trial arms consisted of standardised antiemetics plus either acupressure wristbands, sham acupressure wristbands or antiemetics alone. In the true acupressure group patients were provided with a pair of wristbands. These bands are elastic wristbands with a 1 cm protruding round plastic button, which presses on the P6 acu-point. The sham wristband group received identically appearing wristbands, with the only difference being that they had a flat button in place of the protruding button, thus exerting no pressure on the P6 acu-point. Those allocated to receive wristbands were instructed to wear them throughout the first 7 days of 4 cycles of chemotherapy. Primary outcome assessment was the Rhodes Index for Nausea and Vomiting [18] which was carried out daily for 7 days per chemotherapy cycle. Additional assessment tools which were completed during each cycle of chemotherapy included the MASCC Antiemesis Tool (acute and delayed) [19], hospital anxiety and depression (HAD) scale [20], EQ-5D Utility scale [21] and the FACT-G quality of life scale [22]. At baseline participants also completed measures of nausea expectation, and expectations from using acupressure wristbands. An economic evaluation was carried out based on drug and health service utilisation from the perspective of the health and social care provider.

### Sample

A convenience sample of 26 patients volunteered to participate in the qualitative study to explore their experiences of using acupressure wristbands, and taking part in the clinical trial. Participants were recruited from each of the three geographical sites from which the trial was conducted. Nine were recruited from Manchester, 9 from Liverpool, and 8 from Plymouth and the surrounding regions. Ten of the participating patients had received true acupressure during the trial, 9 sham acupressure, with 7 receiving no acupressure. Sixteen participants were diagnosed with breast cancer, 6 colorectal, 3 lung, and 1 Non Hodgkin’s Lymphoma. Nine of the sample were classified as having received chemotherapy of high emetogenic potential, 12 moderate, and 5 low. Participants had a diverse range of expectations and demographics. The age range of the participants was 35–79 (mean 55, median 55), 7 were male and 19 female. Expectation of how much nausea participants expected to experience during their chemotherapy treatment ranged from 0–10 (mean 5.8, median 5), with 0 being 'not at all’ and 10 being 'very frequently.’ While participants’ belief that acupressure wristbands would help manage their sickness ranged from 2 to 10 (mean 6.9, median 6), with 0 being 'not at all’ and 10 being 'will help me a lot.’

## Methods

A constructivist epistemological approach was adopted for the qualitative study based on the belief that, “data do not provide a window on reality. Rather, the 'discovered’ reality arises from the interactive process and its temporal, cultural, and structural contexts” [23]. Invitations to participate in a qualitative interview were posted to all patients who completed their participation in the clinical trial of acupressure wristbands, or withdrawal from the trial, between February 2010 and December 2010. Interested patients returned a contact sheet to members of the research team who subsequently telephoned them, discussed the qualitative study, obtained verbal consent, and arranged a face-to-face interview. All patients willing to participate were interviewed. All interviews were conducted within 3 months of patient's completing, or withdrawing, from the trial. In-depth semi-structured interviews were conducted with the participants in their own homes or other location convenient for participating patients. The interviews were preceded by obtaining written consent. Interviews were conducted by three members of the research team (JH, WR, MB) and were directed by a topic guide. The topic guide was devised by the researchers based on previous research exploring experiences of receiving acupuncture or participating in clinical trials. The topic guide incorporated a series of open ended questions designed to explore participants’ experiences of taking part in a trial of acupressure wristbands, and for those allocated to receive wristbands, their experiences of using them. Topic guides were updated throughout the study to incorporate emerging themes. Interviews were audio-taped and transcribed verbatim. Interviews lasted for between 30 and 70 min. Transcripts were analysed thematically using Framework analysis; a manual, matrix method, which facilitates thematic and cross-case interpretation [24,25]. Framework analysis was the chosen method for data analysis as this provides a transparent five step approach and provides a clear process for cross-referencing and comparing themes across participants. Analysis proceeded in five stages:

• Familiarization. Transcripts were read and re-read by members of the research team to familiarize and immerse in the data.

• Identification of the thematic framework. Key issues, concepts and themes arising from the data were identified by the research team, and grouped thematically to construct a conceptual framework.

• Indexing. Two of the research team (JH, WR) independently applied the thematic framework to the same transcript to explore any differences in application. The thematic framework was then applied systematically to all the data (JH).

• Charting. Thematic matrices were constructed for all identified categories/subcategories to further summarise and synthesise the indexed data (JH). Thematic matrices consisted of tables of well-defined units of text, with identified categories and subcategories along the x axis and individual participants along the y axis.

• Detection, categorization and classification. The original research questions were reconsidered, and the charts examined in order to define concepts, map the range and nature of phenomena, find any associations and provide explanations (JH).

## Results

Major categories and sub-categories describing participants’ experiences of taking part in the trial and wearing the acupressure wristbands emerged from the data:- *views on complementary therapies*; *deciding to participate*; *experiences during trial participation*; *wearing wristbands*; *perceptions of wristband effects* and *placebos in research*. The category *placebos in research* will be explored in a separate publication as the category was not linked to participants’ experiences of receiving the intervention or participating in the clinical trial.

### Views on complementary therapies

Most participants had only limited personal experience of receiving complementary therapy treatments prior to taking part in the trial of acupressure wristbands. Despite limited personal use of complementary therapies, a number of participants expressed an awareness of the current lack of unequivocal research into the evidence base underpinning many complementary treatments. Participants frequently described themselves as 'open minded’ with regards to complementary therapies and their effectiveness. Interviewees often cited the successful treatment of family/friends, or the long history of some of the treatments, with comments such as 'it’s been around for years, so there must be something in it’ being common. Complementary therapies were also perceived as being 'natural’, 'safe’ treatments associated with fewer side effects than conventional medicine. Interestingly, some participants indicated that their involvement in the trial had altered their perception of complementary therapies. Typically, their personal experiences of using the acupressure wristbands resulted in them having a greater conviction in the benefits of complementary therapies.

“Because I mean you do realise that you know if you take medications you erm… yes if you take the drugs and that basically, you know you take one drug for one thing and then you probably got to take another one to counter act that one, whereas the erm… the more natural remedies, you know I don’t think is probably quite so bad.” [48 year old female receiving high emetogenic chemotherapy allocated to receive no wristbands during the trial].

“The complementary therapies are holistic and they’re actually…a lot of the things they’re using have been, you know, handed down for generations.” [58 year old female receiving low emetogenic chemotherapy, allocated to receive sham wristbands].

Most participants saw the role of complementary therapies as being in combination with conventional medicine. Indeed some participants indicated that they would only be willing to receive complementary therapies if they were provided within the National Health Service (NHS), or if they were advised to use them by a treating conventional healthcare practitioner. Irrespective of whether participants had any previous experience of using complementary therapies, they were near unanimous in the importance which they placed on research into the effectiveness of complementary treatments. There was a view amongst some, that there should be a greater integration of complementary therapies and conventional therapies provided within the NHS, but that this increasing integration should be underpinned by an emerging evidence base.

“I think it’s very important [research into complementary therapies]. Yes. Because I see complementary medicine as being non-invasive being, as being an aid to conventional medicines, or an add-on to conventional medicines. Yes.” [57 year old female receiving high emetogenic chemotherapy, allocated to receive sham wristbands].

“It is something in general complementary medicine is something that are never taken seriously, I don’t think, but they should be because you know you hear some remarkable stories about them, so I think they should be up there with all the drug research and everything yes, definitely.” [59 year old female receiving moderate emetogenic chemotherapy, allocated to receive true wristbands].

### Deciding to participate

Interested patients were provided with details of the study and what participation would involve and were required to consider participation for at least 24 hours before consenting. While many discussed participation with their clinical team or significant others, for the majority of participants the decision to take part in the trial appeared to have been made almost immediately, with phrases such as 'I didn’t hesitate’ being common.

When asked about their reasons for participating in the clinical trial, participants identified a range of factors influencing their decision. Undoubtedly the main motivational factor influencing participants was a desire to 'give something back’. There was an appreciation that the willingness of previous patients to engage in research had improved their own cancer treatment, and that for cancer treatments to improve in the future current patients would need to take part in research studies. In particular, it was the altruistic act of attempting to improve the care of future cancer patients which motivated participants to assist in evaluating if acupressure wristbands were effective for chemotherapy related nausea.

“My reasons were as I’d had various treatments obviously associated with the diagnosis I’d had earlier that year it was partly that each time I had treatment I did reflect on people that had gone before me who from their experience and their participation in possible research that that perhaps made things easier for me in my experience. So it was a small way of trying to help for the future. That was kind of in my head, that I just wanted to try and be part of this and hopefully that would help people like me in the future.” [55 year old female receiving moderate emetogenic chemotherapy, allocated to receive no wristbands].

“Just to assist in any way I could with any product or any medicine or anything that might help people in the future. That was basically it.” [59 year old female receiving moderate emetogenic chemotherapy, allocated to receive true wristbands].

Participants were aware that their chemotherapy treatment was associated with a number of side-effects, including nausea and vomiting. Participation in the trial did not affect antiemetic prescribing as randomization to receive wristbands was offered in addition. A desire to limit their own experience of nausea and vomiting during their chemotherapy treatment was also a strong motivational factor for almost all participants.

“Because I could see all the list of things that you have, the after effects, you get tummy upsets and stuff like that. Then I thought well, if that’s going to help me I don’t want to keep being sick, if it does help me I’ll have a go.” [79 year old male receiving low emetogenic chemotherapy, allocated to receive true wristbands].

“One of the biggest concerns I had when I was approaching having chemotherapy was purely based on what you hear from other people, and nausea kept coming up all the time. So, again, I thought well maybe there would be some benefits as well for me, because obviously that’s what the research was about.” [55 year old female receiving moderate emetogenic chemotherapy, allocated to receive no wristbands].

A minority of participants identified additional motivational factors. These included the lack of any perceived risk of adverse effects from taking part in the trial, and a preconceived belief in the effectiveness and value of complementary therapies. For participants attending a hospital in one of the study sites, the dialogue with their medical consultant was also identified as influencing the decision of some patients to participate. Specifically, information regarding additional funding which the hospital itself would receive from patients participating in clinical trials was an extra motivational factor.

“It was very clear wasn’t it from the beginning that there wasn’t going to be any adverse side effects from it, because I don’t honestly think I would have done it if there were given the fact that I was going to be, have so many side effects from the chemotherapy you just wouldn’t want to add any more adverse side effects to the ones you were already having at the minute so, yes” [44 year old female receiving high emetogenic chemotherapy, allocated to receive true wristbands].

“Yes he was very supportive [hospital consultant] and he did ask me every time I went for an appointment how it was going. And he explained as well that in addition to finding out whether it worked it also provided additional funding into the team, so that just reinforced that I wanted to do it really.” [38 year old female receiving high emetogenic chemotherapy, allocated to receive sham wristbands].

### Experiences during trial participation

Only a couple of participants had any previous experience of taking part in a research study prior to the acupressure wristband trial. Participants indicated they felt they had a good understanding of the study before providing their written consent, with many recounting the written and verbal information they had received before consenting. Part of this process was information on the randomisation process, and participants were pragmatic in their perception of randomisation, exemplified by one female participant who commented 'you are either going to be lucky if you get the wristbands and it might help, you are unlucky you don't get anything and you just carry on as you would do if you wasn’t on the trial.’ However, despite this approach many allocated to treatment as normal still indicated they felt 'disappointed’ at not receiving wristbands.

Many participants expressed initially feeling daunted upon receiving the packs of outcome measures to be completed during the trial. As one female participant stated, 'I thought oh crumbs [laughter]. I thought oh God every day.’ However, these feelings typically subsided once participants familiarised themselves with the outcome forms, and realised that forms were extensively duplicated. Participants generally felt included questionnaires were easy to follow, with comments like 'pretty straight forward’ being common. Although perceived as being easy to complete a few participants felt they were a little onerous, making comments such as 'they were perhaps, perhaps a little bit too lengthy maybe.’ Some indicated that they failed to complete all the paperwork on the designated days. In particular lapses in memory, or poor health as a result of their chemotherapy treatment, led to forms sometimes being completed retrospectively. In some instances this could involve forms being completed up to one week after the date they were supposed to be completed on.

“I think if you start off every night you are doing it, or whatever it is, I will hold my hands up and say, then you kind of, oh I didn’t do it that night, I didn’t do it that night, and then you say I have got to do them forms, and you would rely then on memory. Obviously it weren’t going back weeks, but instead of that, you would start like a bull at a gate every night yes, but you would quickly change to, oh I will blast this all in one go. Probably not right, but that’s being honest with you, then that is what happened and I would think most people do the same.” [41 year old female receiving high emetogenic chemotherapy, allocated to receive no wristbands].

“Some of the days when I was really, really poorly with the chemo I couldn't even get out of bed to even fill it in I’ll be honest, but I knew each day how bad I was, how sick I was. So, you know, was able to fill it in accurately because I knew that at the beginning I was so poorly and so sick and by the end of it you are kind of coming round, so I knew and as soon as I come round and I was out of bed I filled it in.” [49 year old female receiving moderate emetogenic chemotherapy, allocated to receive no wristbands].

Participants expressed satisfaction with the organisation and running of the acupressure wristband trial. A number felt that they had experienced positive outcomes as a result of taking part in the trial and this linked to their reports of motivations for taking part. Most frequent was a sense of wellbeing as a result of feeling they had completed an altruistic act and helped others. Additionally, some felt that the process of completing the trial outcome measures had been of benefit to them. Specifically, that it provided them with some control, at a time when most felt a lack of control over their cancer experience; or that they gained benefit from being able to reflect on how their symptoms had improved during previous cycles of chemotherapy. In contrast a small number of participants indicated they had experienced some negative effects from completing the outcome measures, chiefly that reading and rating their level of nausea and vomiting at a time when they were feeling nauseous had at times worsened their experience.

“I think taking part in the trial is quite, it makes you feel better actually because it is a useful tool and it’s going to be of use for other people in the future…… Yes because it makes you feel better doesn’t it if you feel you are contributing something.” [53 year old male receiving moderate emetogenic chemotherapy, allocated to receive no wristbands].

“I just completed the forms as requested and it was no hassle at all. And actually it helped because it was something positive to do, on certain days and ticking the boxes and all that sort of thing, I felt because I think part of having cancer is you lose control, and I am quite, the sort of person that likes to be in control and this is enabling me a little bit of control back, so that part I quite enjoyed actually.” [55 year old female receiving high emetogenic chemotherapy, allocated to receive sham wristbands].

### Wearing wristbands

Those participants who were allocated to receive either true or sham wristbands during the trial were asked about their experiences of using them. Almost all participants interviewed appear to have worn the wristbands as instructed, keeping them on for 7 days following chemotherapy and only removing them when washing or bathing. Indeed some participants continued using the wristbands after the 7 day period, while many also continued to wear the wristbands after the 4 chemotherapy cycles they participated in the trial. Participants were asked to demonstrate how they wore the bands, with all but two of those interviewed apparently wearing the wristbands in the correct position during the trial. The two participants who were found to have worn the bands inappropriately had both been allocated to receive sham wristbands. In one instance the participant wore the wristbands inside out (meaning slight pressure would have been applied to the P6 acupoint); while in the other the participant indicated that they had manually applied pressure to acupoint P6 during the trial.

Participants reported that they had not experienced any restrictions from wearing the wristbands in terms of everyday activities, other than washing and bathing. As one female participant commented, 'I don’t have many household chores, but no not at all, not at all. I just, take them off when I have a bath or a shower, other than that no they are not intrusive at all.’ For most participants the wristbands were seen as comfortable to wear. Although a few participants reported that they had experienced minor irritations, such as the wristbands feeling tight or painful, or their wrists becoming itchy. Reported adverse side effects were generally deemed minor and acceptable.

“I find sometimes my, it gets, my skin gets quite red, and they rub.” [35 year old female receiving moderate emetogenic chemotherapy, allocated to receive sham wristbands].

“I did find the bobble that went into your arm painful a few times.” [56 year old female receiving high emetogenic chemotherapy, allocated to receive true wristbands].

A number of participants highlighted the fact that they had been questioned by other people about wearing the acupressure wristbands. For many, the curiosity of others had little influence on band use, as one male participant commented, 'if they don’t like it, that’s their problem….. didn’t influence me whatsoever.’ However, a few revealed that they had inhibitions about wearing them in the company of others; or had received negative responses from others to their wearing of the wristbands. When asked about this, some compared the minor impact of wearing acupressure wristbands to the more outwardly visible consequences of their conventional medical treatment, such as loss of hair or having surgical pointers displayed on their body.

“It’s a silly point but they are there all the time, so you do sort of start pulling your sleeves down because you don’t want people to see them, you know it looks rather strange walking round with two wristbands on….. Well everybody knew about them, because I, you know you chat to people and people ask you, and so, I would just tell people but if you went for a meal or if you were out somewhere socially where people didn’t know you, you would feel slightly embarrassed. These two bands, that look as though you should be in the gym.” [57 year old female receiving high emetogenic chemotherapy, allocated to receive sham wristbands].

“They didn’t take any notice of it whatsoever it wasn’t that obtrusive, I am more self conscious about the hole in my throat, than I were of any bloody arm bands. So I usually had a coat on anyway and nobody would outside hardly seen them.” [66 year old male receiving moderate emetogenic chemotherapy, allocated to receive true wristbands].

### Perceptions of wristband effects

Almost all participants interviewed had only experienced mild nausea or vomiting during the trial. They were pragmatic about the extent to which the wristbands were responsible for this lack of nausea and vomiting during the trial. They reported that there could be a number of other reasons, including their antiemetic medication, basic constitution, or just luck! However, many participants, including some patients receiving sham acupressure, believed the wristbands to have had a positive impact on their nausea and vomiting; there was a perception that the wristbands were, at least in part, responsible for the lack of nausea and vomiting they had experienced. For some this was due to their experiences during the trial, such as participants who noticed their nausea was greater if they didn’t have the wristbands on, or if they were wearing the wristbands in an incorrect position. The wristbands were not seen as being any more, or less effective, at different times of day, or at different points in the participants’ chemotherapy treatment. There were no additional benefits reported from wearing the wristbands beyond the potential alleviation of nausea and vomiting.

“Until the trial is complete you can’t really say whether it helped you or not can you…. I wore them, yes I wore them each time I had treatment yes. And erm yes I feel as though I benefited from them, and I think I possibly would have been more sick than what I was. I felt the bands did help in that respect. Yes.” [55 year old female receiving moderate emetogenic chemotherapy, allocated to receive true wristbands].

“I would be feeling a bit sick and I would think 'goodness me, why am I feeling so sick’ and then I would put them on and it would improve and one day I was feeling sick with them on, but they weren’t in the right place (laughs) I was looking at them and they weren’t in the right place, so there is a reason you know, this is why I have got such belief in them now.” [56 year old female receiving high emetogenic chemotherapy, allocated to receive true wristbands].

Irrespective of perceptions of effectiveness, almost all participants indicated that they would recommend the wristbands to other chemotherapy patients, or to patients experiencing nausea and vomiting due to other causes. This related to the fact that, irrespective of whether the wristbands were of benefit for nausea and vomiting, they were not associated with having any negative or adverse effects.

“There isn’t a reason not to wear them. Personally yes. So, yes I would recommend them yes.” [38 year old male receiving moderate emetogenic chemotherapy, allocated to receive sham wristbands].

“It’s not going to harm you in any way, just might help you out, yes, oh yes I would recommend it to anybody going through chemo because I am not sure just how rough I would have felt having the chemo if I didn’t have the bands.” [56 year old female receiving high emetogenic chemotherapy, allocated to receive true wristbands].

## Discussion

This nested qualitative study aimed to explore cancer patients’ experiences of using acupressure wristbands, and taking part in a randomised controlled trial for a CAM intervention. The two main motivators which patients identified for participating in the trial of acupressure wristbands appear largely congruent with the findings of previous research within the fields of cancer and CAM. Nurgat et al. conducted a questionnaire survey to examine the motives of patients entering clinical trials of novel cancer therapies and found that the two main motivational factors influencing participation in a clinical trial were a possible health benefit for the patient themselves and to help future cancer patients [26]. However, of these the authors found patients appeared to be primarily motivated by a personal health gain, a finding which is consistent with other published research within cancer [27,28]. These were again found to be the two main motivators for patients participating in the trial of acupressure wristbands. Contrary to previous work of Nurgat and others [26-28], 'giving something back’ appeared a stronger motivational factor than personal health gain. Two nested qualitative studies have explored the motivations of patients participating in clinical trials of acupuncture. In the first patients’ felt acupuncture was 'worth a try’, with patients being eager for symptom relief and to help with research for altruistic motives [16]. While in the second study patients were found to be primarily motivated by a sense of curiosity and for altruistic reasons, although a smaller number of patients were identified as being motivated by a desire for symptom relief [14].

Patients were largely satisfied with the organisation and running of the acupressure wristband trial. The process of recruitment was generally perceived as straightforward and had been adequately described. Interestingly, a number of patients experienced positive outcomes as a result of participating in the acupressure trial. These included a sense of wellbeing from the altruistic act of helping future cancer patients, as well as the process of completing trial paperwork providing some control and a means for patients to reflect on how their symptoms had improved. This is a finding consistent with those of previous research, with other studies reporting that trial participants frequently perceive benefits from participating beyond any specific effects of the interventions administered, and including aspects of the participatory process itself. These have included patients obtaining beneficial effects from helping others through participating in research [29], and insights gained through completing trial diaries of their symptoms [16]. A greater awareness and reporting of these secondary beneficial outcomes for patients participating in clinical trials could have positive implications for recruitment to future clinical trials within cancer.

The primary outcome assessment for the clinical trial of acupressure wristbands was level of nausea. Analysis of the trial data revealed no statistically significant differences between the three groups (true wristbands, sham wristbands, and no wristbands) [17]. However, the median nausea experience in patients using wristbands (both real and sham) was somewhat lower than that in the antiemetics only group [17]. The qualitative data from the present study indicates that patients in both the true and sham wristbands arms typically attributed the wristbands as having had a positive impact on the low levels of nausea and vomiting experienced during the trial, with participants indicating they would recommend the wristbands to other cancer patients receiving chemotherapy. The quantitative and qualitative data suggest that patients derive some benefit from using the wristbands, irrespective of whether they are true or sham. It has been suggested that CAM interventions may be associated with eliciting enhanced placebo responses [30]. The findings from the present qualitative study and clinical trial of acupressure wristbands would appear to suggest that any benefit experienced by patients was likely to be the result of a placebo effect. However there is considerable debate regarding the conceptualisation of placebo effects. In particular whether the placebo effect should be viewed as a therapeutic physiological response which could be studied and harnessed to improve outcomes in patients [30-33]. It has been argued that the placebo effect should even be redefined as a 'meaning response’ [34]. Certainly recent qualitative research suggests that knowledge that the efficacy of CAM interventions could be based, to a greater or lesser extent, on a placebo effect does not necessarily undermine their appeal to patients [35].

Health service providers are placing increasing significance on the experiences and preferences of patients. Published research indicates that providing patient-centred, tailored care can have a positive influence on various health outcomes [36]. These include mortality [37], health behaviour [38], treatment adherence [39], and self management [40]. Although the findings of our own trial found acupressure to be ineffective for chemotherapy related nausea, some previously published trials have found acupressure to be effective [41]. It would therefore appear advisable that any future decisions relating to the health service provision of acupressure wristbands include the findings from qualitative research exploring patients’ experiences in combination with all relevant economic and clinical trial data.

Previous qualitative research exploring the experiences of patients receiving treatment with acupuncture suggests patients experience a range of benefits beyond the alleviation of their presenting condition, including improvements in overall wellbeing, sleep pattern, and energy levels [1-6]. Many of the patients allocated to receive wristbands who participated in interviews in the present study perceived these wristbands, both true and sham, as having a positive impact on the level of nausea and vomiting they experienced. However, none of the patients who participated in interviews associated the acupressure wristbands as eliciting benefits beyond the relief of chemotherapy related nausea and vomiting. This may suggest that a greater stimulation of acupuncture points is required to elicit these expanded effects; or that the contextual factors within the acupuncture consultation, such as the interaction between the practitioner and patient, or the consultation setting, may work in conjunction with the stimulation of acupuncture points to elicit these expanded effects of care [42].

The findings from the present study have ramifications for the validity of the findings of the acupressure wristband trial [17], and more generally the future design of clinical trials within the field of cancer. The data from the present nested qualitative study revealed that two of the nine patients receiving sham acupressure who were interviewed had worn their wristbands inappropriately, with both consequently receiving active treatments. The study also revealed an apparent delay in some patients completing the trial paperwork, relying on memory to complete outcome forms during the trial. Both of these may represent confounding variables, which if generalisable to other patients in the acupressure wristband trial, could have serious implications for the results of the trial, and may be a factor in the trial finding the wristbands to be ineffective [17]. Cancer and its conventional treatment are associated with considerable symptoms and a decline in physical function [43,44], and the findings of the present study highlight the difficulties which patients experience in completing trial outcome forms during this period. It is therefore advisable that future clinical trials consider this when designing their trial protocol and select outcome assessments which will reduce the potential burden on patients and improve the likelihood of patients completing the assessments on the designated day(s).

It should be noted that as participants self-selected to take part in qualitative interviews, this may represent a potential limitation of the study. Specifically, it cannot be ruled out that those patients who believed they had benefited from using the acupuncture wristbands, both real and sham, may have been more likely to agree to participate in an interview exploring their experiences than those patients who did not experience any benefit from wearing the wristbands.

## Conclusions

Qualitative studies nested within clinical trials have been underemployed within the field of CAM. The present study demonstrates the potential value of qualitative research of this nature. The fact some patients wore wristbands inappropriately, and/or delayed completion of trial paperwork are potentially serious threats to the validity of the trial findings, and bring into question the conclusions from this trial [17]. In addition the research provides an insight into cancer patients’ motivations for, and experiences of, taking part in a clinical trial for a CAM intervention. This includes benefits to the patients themselves from the participatory process of taking part in the trial. In addition the research also represents the first qualitative study to explore patients’ experiences of using acupressure wristbands and perceptions of effects, with patients’ perceiving the wristbands as reducing the level of nausea and vomiting experienced during chemotherapy treatment.

## Competing interests

The authors declare they have no competing interests.

## Authors’ contributions

AM conceived of the study; all authors were involved in the design of the study; JH, WR and MB carried out the qualitative interviews; JH analysed the qualitative data and led on preparing the manuscript; all authors contributed to the manuscript and read and approved the final version.

## Pre-publication history

The pre-publication history for this paper can be accessed here:

http://www.biomedcentral.com/1472-6882/13/260/prepub
